# Precision Grip Control while Walking Down a Stair Step

**DOI:** 10.1371/journal.pone.0165549

**Published:** 2016-11-01

**Authors:** Daniela Ebner-Karestinos, Jean-Louis Thonnard, Yannick Bleyenheuft

**Affiliations:** 1 Institute of Neuroscience, Université catholique de Louvain, Brussels, Belgium; 2 Cliniques Universitaires Saint-Luc, Physical and Rehabilitation Medicine Department, Université catholique de Louvain, Brussels, Belgium; University of Chicago, UNITED STATES

## Abstract

The aim of this study was to determine whether the internal model regulating grip force (GF)/load force (LF) coordination during a brisk load increase is preserved when the lower extremities produce a perturbation during a single step-down task. We observed the coordination of the vertical ground reaction force (vGRF), GF and LF while holding a handheld object during a single step-down task. The 3 forces (vGRF, GF and LF) decreased during the start of the task. While the subject was descending, LF and GF became dissociated from vGRF and increased in value, probably to anticipate the first foot contact. Coordination of LF and GF was maintained until the maximal vGRF (knee extension). LF peaked in the same time window as vGRF, whereas GF peaked about 70 ms later. This desynchronization, which was previously observed in direct load increase on a handheld object, was interpreted to be a predictive action to ensure the smooth management of the brisk increase in load induced by the lower extremities. Incidentally, in this group, kinematic and dynamic differences were observed between men and women, which may highlight a gender-specific strategy to perform the step-down task. In conclusion, these results suggest that the internal model of precision grip is able to integrate a brisk load change, whatever its origin, and regulate the forces to provide an ideal GF to dampen a brisk load increase and secure the object.

## Introduction

The manipulation of small objects between the thumb and index finger requires the precise coordination of grip force (GF, perpendicular to the object) and load force (LF, tangential to the object) in order to avoid inadequate forces that may induce the crushing or the slippage of the handheld object [1]. The concomitant adjustment of the GF to the changes in the LF–generated by the movements of the object–requires the use of a predictive model. This model allows GF to anticipate LF changes in order to provide a parallel change in the GF and LF [2]. The precise coordination of forces is thus adapted to the characteristics of the object (e.g., shape, weight, contact surface [1]) and its forthcoming movements, predicting changes in forces and updating this prediction [3]. This has been notably evidenced during adaptations to different weights and textures, as well as in studies without cutaneous feedbacks. By pseudo-randomly changing the weight of the handheld object during lift-hold-replace tasks, Westling and Johansson established that the GF changed in proportion to the weight of the object. In the same experiment, they varied the frictional condition between the fingers and the object by changing the grip surfaces, evidencing an increase in GF with more slippery surfaces [4]. Further evidence of a predictive model taking into account the cutaneous inputs from the fingers was provided by object manipulation in cyclic movements under anesthesia [5]. A decline in GF was observed during the task, likely due to the lack of integration of this cutaneous information in the predictive model of manipulation [5].

More recently, studies of precision grip focused on the grip-load coordination during brisk load changes [3, 5–8]. While the subjects grasped an instrumented object with a precision grip, a brisk increase in load was induced by the drop of a mass attached to the instrumented object. The drop was either self-induced by the participants or unexpectedly induced by the examiner [6, 7], or no impact occurred [3]. These studies demonstrated that prior to the brisk increase in load the GF started to increase aiming to anticipate this change in LF. After the brisk load increase, the GF encountered a second increase and reached a maximum in a window of ~170ms after the brisk load increase [3, 6, 7]. While initially hypothesized as a reactive component, the GF occurring after brisk load increase was evidenced as a predictive mechanism [3]. This was particularly evidenced, as this GF increase after load change was still present when a brisk change was expected but did not occur. This anticipatory mechanism was also studied in collisions induced by a pendulum on a handheld object. A GF increase after the impact was also observed, highlighting the predictive nature of the grip mechanism [8–10]. In addition, the GF/LF adaptation to brisk changes has been studied in altered gravity while performing collisions in a point-to-point task [11–13]. These studies highlighted a maximum GF delayed regarding the maximum LF generated by the collision, which was explained as a predictive mechanism aiming to damp the collision and secure the grasp subsequently [11–13]. Brisk changes in load induced by a brisk increase in the object mass, a collision generated on the object by a pendulum, or by the object colliding a static support are thus systematically generating an anticipatory control including 1) a GF increase prior to the load increase, and 2) a GF increase afterwards. However, all these experiments were performed in a static sitting position. It is unknown how the GF/LF coupling would adapt if a brisk load change was generated by an event arising in lower extremities.

In everyday life, rapid changes in load on a handheld object can be induced during locomotion (e.g. holding a cell phone or cup of coffee while going down a step). While walking and moving forward, the cyclic vertical movement of the body and the upper extremities may affect the inertia of a hand-held object [14]. Explorations of object transport during gait showed a synchronization between the forces exerted by the hand on a handheld object (grip/lift coupling) and the cyclic task performed by the lower extremities [15–18]. Indeed, in these studies, maximum values of the forces were observed concomitantly, shortly after the moment of floor contact, when the handheld object was at its lowest vertical position [16, 17]. Minimum force values appeared at midstance (one leg support), when the object was at its highest position in the sagittal plane [16].

To our knowledge, upper/lower extremity coordination during rapid load changes (e.g. going down a step) has never been studied. This coupling of upper and lower extremities is of interest to define how intersegmental coordination is controlled in a discrete lower extremity task, in which the cyclic aspect of movement is disrupted. Going down a step induces a brisk change in dynamics, which is likely to influence the control of precision grip needed to hold an object. Therefore, the aim of this study was to assess the ability of the predictive mechanisms underlying GF control to adapt to a perturbation generated by lower extremities, namely, walking down a single step. In a secondary analysis, we explored the possibility of gender differences during this discrete motor task. We hypothesized that the predictive model of precision grip will be able to integrate the brisk perturbation generated by the lower extremities. The grip-lift coupling will be maintained during this intersegmental task, with load force adapting to vGRF and grip force adapting to load force.

## Method

### Participants

Twelve healthy participants (6 women, 6 men; mean age 29.2 ± 4.0 years; mean height 169.4 ± 7.5 cm; mean weight 66.0 ± 13.2 kg) presenting right hemi-body dominance participated in this study. The study was conducted under approval of the Ethical Committee of the Université catholique de Louvain. All participants provided written informed consent.

### Materials

An Elite system (ELITE-BTS, Milan, Italy) with eight infrared cameras was used to track the three-dimensional (3D) position of two reflective markers (on the sacrum and on a handheld object) at a sampling rate of 200 Hz. A force platform (220 × 80 cm) recorded the ground reaction force by four 3D strain gauges at a sampling rate of 1000 Hz [19]. We considered only the vertical component of the ground reaction force (vGRF), which we calculated as the sum of the vertical component of the force captured by the left and right strain gauges located at each of the corners of the platform [20].

**Fig 1:**
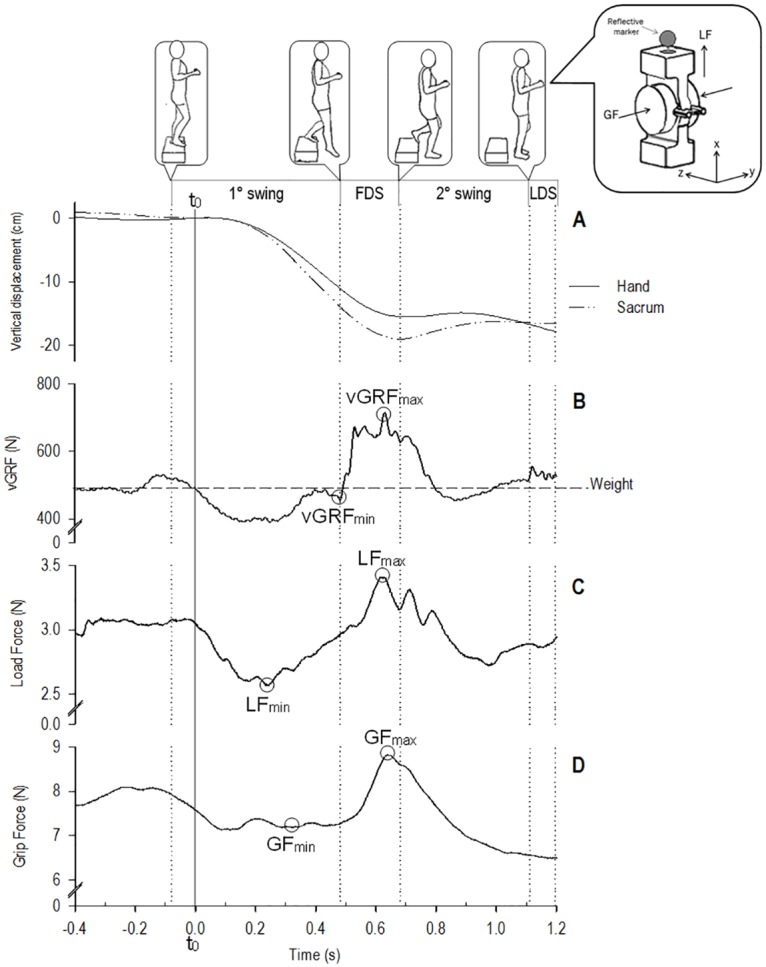
Example of typical recordings of the step down task in one participant. Top panel: phases of the task (first swing, first double support (FDS), second swing and last double support (LDS)) with images corresponding to the initial position of each phase. Inset: Isometric (posterior left) view of the Grip-Lift Manipulandum (GLM). The direction of the grip force (GF) and load force (LF) are shown by its vectors following the reference frame where x, y and z corresponds to the vertical, mediolateral and posteroanterior axes respectively. In the panels, the following traces are shown as function of time: (A) vertical displacement of the hand and sacrum, (B) vertical Ground Reaction Force (vGRF), (C) Load Force (LF) and (D) Grip Force (GF). The short vertical dotted lines mark the different phases of the task in all traces. The straight long vertical line mark t_0_ in all four traces. The dashed horizontal line in panel B shows the subject weight. The circles highlights the events observed in the forces during the task: vGRF_fc_, value of the vGRF at foot contact; vGRF_max_, maximum value observed for vGRF during the task; LF_min_, minimum value observed for LF during the task; LF_max_, maximum value observed for LF during the task; GF_min_, minimum value observed for GF during the task; and GF_max_, maximum value observed for GF during the task.

We employed a 290-g Grip-Lift Manipulandum (GLM; Arsalis), with dimensions of 91 × 37.5 × 48 mm and grip aperture of 43.2 mm (Fig 1, inset). The GLM was equipped with two (right and left) 3D Mini-40 force and torque sensors (ATI Industrial Automation, Apex, NC, USA), used to measure the precision grip forces. This device calculates LF (total tangential force applied on the object) and GF (perpendicular force applied on the object), on the basis of three force components (F_x_, F_y_, F_z_) captured by each sensor. LF was calculated as LF = LF_right_ + LF_left_, where $\text{L}{\text{F}}_{\text{i}}=\sqrt{{\text{F}}_{\text{x}}^{2}+{\text{F}}_{\text{z}}^{2}}$ for each sensor (i = right, left). GF was calculated as $\text{GF }=\;\frac{{\text{F}}_{\text{y},\text{r}}-{\text{F}}_{\text{y},\text{l}}}{2}$, where r and l correspond to the right and left sensors, respectively. Each acquisition had a sampling frequency of 1000 Hz.

### Procedure and experimental protocol

The task was described to each participant. Participants stood barefoot on a wooden step (17 × 61 × 30 cm). From a static standing position, participants were asked to go down the step and maintain a static bipedal ending position (Fig 1, top panel). The left arm was free along the body, while the right elbow was flexed about 90°, with the GLM being held in the right hand between the thumb and index finger. Each participant performed the step-down task eight times.

After the task, we measured the friction between the fingers and the GLM and the maximal precision grip force for each participant. Friction was measured by asking the participant to release the precision grip force gradually until a slip occurred, for three consecutive trials. The static coefficient of friction (CoF) was computed as half of the LF/GF ratio at slip onset. The mean CoF was estimated for each subject. The maximal force was measured by asking the participant to pinch the GLM with a maximal force during three consecutive trials, with a 1-min rest between each trial.

### Data acquisition and analysis

Four different phases were observed in the task based on the description of gait control while descending stairs made by Zachazewski, Riley and Krebs (1993) [21] (illustration in top panel of Fig 1): the first swing, the first double support (FDS), the second swing and the last double support (LDS). The *first swing* begins when the right foot rises from the step and lasts until the foot contacts with the floor, initiating the *FDS*. Subsequently, the *second swing* starts with the rise of the left foot from the step until it contacts the floor, initiating the *LDS*. Our analysis focused on the first three phases (first swing, FDS and second swing). During these phases, six *events* were considered as essential for subsequent analysis: foot contact at the start of FDS and maximal vGRF after foot contact (Fig 1, vGRF_fc_ and vGRF_max_); minimal and maximal LF (Fig 1, LF_min_ and LF_max_), and minimal and maximal GF (Fig 1, GF_min_ and GF_max_).

### Variables

*Kinematic variables* were the vertical displacements of the hand and sacrum (in cm) during each of the six events (Fig 1A). *Dynamic variables* included vGRF, LF and GF at each of the six selected events (Fig 1). Thus, a total of 18 dynamic variables were analysed. In addition, the GF/LF ratio at each event was calculated. The static CoF between the fingertips and GLM was computed as half of the LF/GF ratio at slip onset, detected visually in the force traces:

CoF=(LF at slip/2GF at slip)

Safety margin (SM) was calculated as the difference between GF at each event and GF at slip:

SM=(GFx−GF at slip)/GF at slip

where x corresponds to each event and

GF at slip=(LF at slip/2CoF)

*Temporal variables* were studied according to foot contact at the start of FDS and at maximal vGFR (Fig 1, vGRF_fc_ and vGRF_max_). These two events delimit the brisk load increase in dynamics of the lower extremity, and are likely to have an impact on the forces of the upper extremities. To observe a possible concomitance between upper and lower extremity events, we calculated the delay between the onset of the increase in forces in the hand and the start of FDS (Fig 1: LF_min_ → vGRF_fc_ and GF_min_ → vGRF_fc_), and the peak-to-peak delay between forces. We considered for these delays in each trace the first peak after force increase (Fig 1: LF_max_ → vGRF_max_ and LF_max_ → GF_max_). We assessed the anticipatory delay in forces of the hand to the maximal constraint (Fig 1: LF_min_ → LF_max_ and GF_min_ → LF_max_). A total of six temporal variables were analysed.

### Statistics

One-way repeated-measure analysis of variance was performed to search for a possible effect of repetition over eight trials of the single step-down task. Post-hoc analyses were conducted by Tukey tests. A trial effect was observed in LF (*p* = 0.038), with significant lower mean values on the first trial compared with the other trials. This result shows a learning effect in the task after 1 trial. Therefore subsequent analyses were performed on the mean values of trials 2 to last. Individual mean values were averaged and are presented in tables as the grand mean and SD. Vertical displacements of the hand and sacrum were compared by using a paired *t*-test. The gender effect was studied by using a *t*-test (or Mann–Whitney rank sum test, when the test for normality or equal variance failed) in all variables. To compare vGRF between male and female groups, we normalized this force to the mean weight of the whole group. Differences were considered statistically significant at the *p* < 0.05 level.

## Results

Fig 1 displays a typical trace showing the phases of the single step-down task (top panel), vertical displacements of the hand and sacrum (panel A), vGRF (panel B), LF (panel C), and GF (panel D). T_0_ corresponds to the moment when vGRF equals the body weight during the first swing (Fig 1, vertical line). Table 1 reports the recorded mean and SD values of the kinematic and dynamic variables during the step-down task, while Table 2 reports values of the temporal variables.

**Table 1:** Mean values (SD) of kinematic and dynamic variables at the different events of the task.

Events	Kinematic Variables					Dynamic Variables									
	*Vertical displacement of Hand (cm)*		*Vertical displacement of Sacrum (cm)*		*Paired t-test*	vGRF [N]		Load Force [N]		Grip Force [N]		GF/LF		Safety Margin	
	*Mean*	*(SD)*	*Mean*	*(SD)*	*p value*	*Mean*	*(SD)*	*Mean*	*(SD)*	*Mean*	*(SD)*	*Mean*	*(SD)*	*Mean*	*(SD)*
***t***_***0***_	0.00	(0.0)	0.0	(0.0)	-	631.8	(117.1)	2.8	(0.4)	8.1	(4.9)	3.0	(1.8)	1.9	(1.7)
**LF**_**min**_	4.9	(2.3)	6.8	(2.9)	0.006	580.1	(160.4)	2.4	(0.4)	7.4	(4.2)	3.1	(1.7)	1.7	(1.5)
**GF**_**min**_	5.2	(2.5)	7.3	(3.5)	0.008	590.6	(157.7)	2.5	(0.4)	7.0	(4.0)	2.9	(1.6)	1.5	(1.4)
**vGRF**_**fc**_	8.3	(1.4)	11.1	(1.1)	<0.001	560.1	(152.3)	2.6	(0.5)	7.5	(4.2)	2.9	(1.6)	1.7	(1.5)
**LF**_**max**_	12.3	(2.0)	15.8	(1.8)	<0.001	829.7	(130.3)	3.3	(0.5)	8.1	(4.1)	2.6	(1.3)	1.9	(1.5)
**vGRF**_**max**_	12.7	(2.0)	16.4	(1.6)	<0.001	982.7	(153.5)	3.1	(0.5)	8.1	(4.1)	2.7	(1.3)	1.9	(1.5)
**GF**_**max**_	13.5	(2.0)	16.7	(1.6)	<0.001	799.1	(108.0)	3.2	(0.5)	8.4	(4.1)	2.7	(1.3)	2.1	(1.5)

LF = load force; GF = grip force; vGRF = vertical ground reaction force; min = minimum value of the force; max = maximum value of the force; fc = foot contact; cm = centimeters; N = newton; SD = standard deviation. Paired t-test results were considered significant when p values ≤0.05

**Table 2:** Mean values (SD) of temporal variables of the whole sample during the task.

	Time [ms]	
Temporal delay	Mean	*(SD)*
LF_min_ → vGRF_fc_	101.3	*(63*.*8)*
GF_min_ → vGRF_fc_	92.4	*(63*.*2)*
LF_min_ → LF_max_	226.5	*(51*.*4)*
GF_min_ → LF_max_	217.6	*(57*.*5)*
LF_max_ →vGRF_max_	17.3	*(21*.*2)*
LF_max_ →GF_max_	73.4	*(42*.*2)*

LF = Load force; GF = Grip force; vGRF = vertical ground reaction force; min = minimum value of the force; max = maximum value of the force; fc = foot contact; ms = milliseconds; SD = standard deviation.

### Kinematic variables

To study the relative displacement between the hand and sacrum, we synchronized their vertical displacements at t_0_ (Fig 1A). The vertical downward displacement of the sacrum (range: 6.8 ± 2.9 cm to 16.7 ± 1.6 cm) was significantly larger than that of the hand (4.9 ± 2.3 cm to 13.5 ± 2.0 cm) at the six time events until the end of the second swing (Fig 1 and Table 1). Thus, the vertical path of the hand was systematically smaller than that of the sacrum (all *p* ≤ 0.008).

### Dynamic variables

The vGRF value decreased during the first swing (Fig 1B), remaining under the body weight of the participant from t_0_ until the start of FDS. A sharp increase of vGRF was observed at foot contact (vGRF_fc_, Fig 1), which led to a peak in vGRF (vGRF_max_, Fig 1) corresponding to the charge of the body weight on the right foot. Thereafter, vGRF decreased during the second swing. Mean vGRF values ranged from 560.1 ± 152.3 N at vGRF_fc_ to 982.7 ± 153.5 N at vGRF_max_, corresponding to 86.6% and 151.9% of the mean body weight of the whole group (646.9 N), respectively (Table 1).

LF and GF were synchronized at the start of the task. They initially decreased during the participant’s descent, reaching minima during the first swing (LF_min_ and GF_min_, Fig 1). However, while the body was still moving downwards (see kinematics of the sacrum), the LF and GF values started to increase. During FDS, the GF and LF values continued to increase in parallel until the peak of LF (LF_max_, Fig 1), when these values became dissociated. LF_max_ increased first, in the same time window as the peak of vGRF (vGRF_max_, Fig 1), followed by the peak of GF (GF_max_, Fig 1). Finally, LF and GF decreased during the second swing. Mean values ranged from 2.4 ± 0.4 N to 3.3 ± 0.5 N for LF and from 7.0 ± 4.0 N to 8.4 ± 4.1 N for GF (Table 1). The GF/LF ratio values varied throughout different phases of the task, ranging between 2.6 ± 1.3 N and 3.1 ± 1.7 N. Similarly, the SM showed variations from 1.5 ± 1.4 N to 2.1 ± 1.5 N (Table 1).

### Temporal variables

The delay between the initial increases of the precision grip forces (GF and LF) and the foot contact (vGRF_fc_), showed a mean value close to 100 ms, with the increase of LF slightly preceding the increase of GF in time (< 10 ms). The temporal delay between the increases of GF and LF and the maximal constraint (vGRF_max_) was approximately 220 ms (Table 2). LF_max_ and vGRF_max_ were concomitant (i.e. LF_max_ → vGRF_max_ = 17.3 ± 21.2 ms). Finally, GF_max_ occurred after LF_max_ (LF_max_ → GF_max_ = 73.4 ± 42.2 ms).

### Gender differences

**Fig 2:**
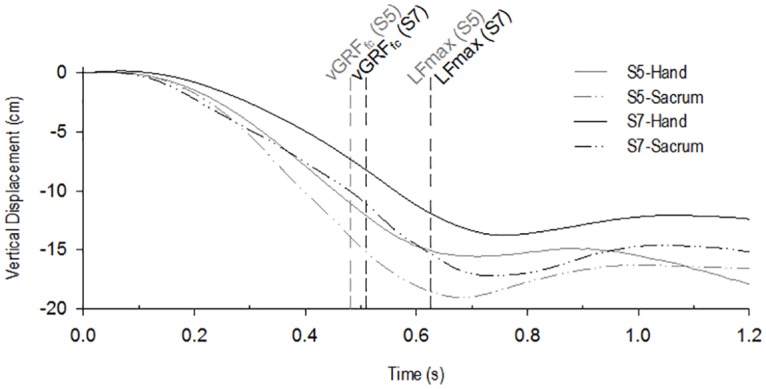
Example of two subjects’ traces corresponding to each groups mean values. Female subject (S5) is presented in grey and male subject (S7) in black. The traces display the vertical position of the hand (straight traces) and the sacrum (line-dot-dot traces) as function of time, starting from t_0_, showing the difference in the vertical displacement of the hands and the sacrum between groups. The vertical cut lines mark vGRF_fc_ and LF_max_ events in each subject’s trial.

Fig 2 shows representative vertical displacements for a woman (subject 5, grey traces) and a man (subject 7, black traces). Traces display the displacements of the hand (straight traces) and sacrum (line-dot-dot traces) as a function of time, starting at t_0_. The female group showed a larger downward vertical displacement in the traces, as was confirmed by the mean values (Table 3). Statistical analysis on the whole sample showed that after the first foot contact, the female group exhibited significantly larger vertical displacements for the sacrum (all *p* ≤ 0.001) and hand (all *p* ≤ 0.05) compared to the male group.

**Table 3:** Comparison of kinematic and dynamic variables between female and male groups.

Event	Group	Kinematic Variables				Dynamic Variables									
		*Vertical displacement of Hand (cm)*		*Vertical displacement of Sacrum (cm)*		vGRF* [N]		Load Force [N]		Grip Force [N]		GF/LF		Safety Margin	
		*Mean*	*t test*	*Mean*	*t test*	*Mean*	*t test*	*Mean*	*t test*	*Mean*	*t test*	*Mean*	*t test*	*Mean*	*t test*
		*[SD]*	*p-value*	*[SD]*	*p-value*	*[SD]*	*p-value*	*[SD]*	*p-value*	*[SD]*	*p-value*	*[SD]*	*p-value*	*[SD]*	*p-value*
**LF_min_**	F	3.7 [2.5–8.0] ^†^		5.4 [3.5–10.9]^†^		540.0 [86.2]		2.5 [0.4]		5.1 [2.0]		2.0 [0.7]		0.7 [0.4–1.7]^†^	
	M	5.0 [2.7–8.3] ^†^	0.685	6.2 [4.5–11.6]^†^	0.412	606.7 [75.0]	≤0.001	2.3 [0.4]	0.271	9.6 [4.8]	0.062	4.2 [1.8]	0.021	1.7 [0.8–3.2]^†^	0.006
**GF_min_**	F	5.8 [3.2]		6.3 [4.9–12.6]^†^		560.9 [522.0–604.4]^†^		2.6 [0.4]		4.9 [2.0]		1.9 [0.7]		0.6 [0.4–1.6]^†^	
	M	5.5 [3.3]	0.788	6.8 [4.8–11.4]^†^	0.843	614.8 [568.9–651.2]^†^	≤0.001	2.3 [0.4]	0.248	9.2 [4.5]	0.058	3.9 [1.6]	0.018	1.7 [0.8–2.9]^†^	0.004
**vGRF_fc_**	F	8.5 [6.8–10.0]^†^		11.4 [1.8]		548.3 [503.4–584.1]^†^		2.8 [0.4]		5.2 [2.0]		1.9 [0.7]		0.8 [0.5–1.6]^†^	
	M	7.7 [7.2–8.3]^†^	0.178	10.7 [1.4]	0.107	589.4 [537.6–608.5]^†^	0.007	2.5 [0.5]	0.318	9.7 [4.8]	0.056	3.9 [1.6]	0.018	1.8 [1.0–3.3]^†^	0.004
**LF_max_**	F	12.9 [2.9]		16.8 [1.5]		879.8 [790.7–1027.3]^†^		3.6 [3.5–3.6]^†^		5.9 [1.8]		1.7 [0.5]		1.0 [0.7–2.2]^†^	
	M	11.3 [2.2]	0.041	14.6 [2.5]	≤0.001	751.6 [685.5–842.1]^†^	≤0.001	2.9 [2.4–3.6]^†^	0.180	10.3 [4.7]	0.061	3.4 [1.2]	0.009	1.9 [1.1–3.4]^†^	0.012
**vGRF_max_**	F	13.2 [2.7]		17.4 [1.2]		1058.6 [948.8–1189.9]^†^		3.4 [3.3–3.5]^†^		5.9 [1.8]		1.7 [0.5]		0.9 [0.7–2.2]^†^	
	M	11.9 [2.0]	0.053	15.2 [1.9]	≤0.001	858.8 [813.2–983.0]^†^	≤0.001	2.7 [2.2–3.5]^†^	0.132	10.2 [4.7]	0.062	3.6 [1.3]	0.006	1.9 [1.2–3.5]^†^	0.015
**GF_max_**	F	13.9 [2.8]		17.3 [16.7–18.3]^†^		855.1 [813.2–935.3]^†^		3.5 [3.4–3.6]^†^		6.2 [1.8]		1.8 [0.5]		1.0 [0.8–2.3]^†^	
	M	12.6 [1.9]	0.057	15.7 [14.5–16.9]^†^	≤0.001	733.4 [689.0–782.5]^†^	≤0.001	2.9 [2.4–3.5]^†^	0.180	10.7 [4.7]	0.053	3.6 [1.2]	0.007	2.0 [1.3–3.5]^†^	0.020

LF = load force; GF = grip force; vGRF* = vertical ground reaction force normalized by the mean weight of the whole sample to compare both groups; min = minimum value of the force; max = maximum value of the force; fc = foot contact; F = female; M = male; N = newton; SD = standard deviation;

^†^ = Mann-Whitney Rank sum test in non-parametric conditions (median & [25%– 75%]). Results were considered significant when p values ≤ 0.05

To compare groups by gender, we normalized vGRF to the mean weight of the whole group. A gender effect was systematically observed in vGRF. Until the first foot contact (vGRF_fc_), the values were systematically higher in the male group (all *p* ≤ 0.007). After the first foot contact, this difference was reversed, with vGRF values being systematically higher in the female group (all *p* ≤ 0.001; Table 3). No gender difference was observed for the LF values.

The male group showed a systematic tendency towards higher GF values (*p* values between 0.053 and 0.062). The GF/LF ratio highlighted a significant systematic difference between men and women, with men presenting higher ratios in all events (all *p* ≤ 0.021). All SM values were significantly higher in the male compared to the female group (Table 3, Mann–Whitney rank sum test, all *p* ≤ 0.020). There was no difference in CoF values between the male and female groups (mean CoF: 0.516 ± 0.2, Mann–Whitney rank sum test, *p* = 0.095). No gender difference was observed for temporal variables.

## Discussion

Brisk changes in force while manipulating a handheld object have been studied in different contexts, including active [12], passive [3] and pendulum-induced [9] collisions. Although these studies highlighted the type of control (essentially predictive) and the mechanisms underlying collision damping, all of these experiments were done in a sitting posture. In everyday life, brisk changes in dynamics may be induced through the lower extremities, such as when jumping an obstacle while holding an object. In this study, we aimed to assess the ability of predictive mechanisms underlying grip force control to adapt to a perturbation generated by the lower extremities, such as walking down a single step. We hypothesized that the grip-lift coupling would be maintained during this intersegmental task, with load force adapting to vGRF and grip force adapting to load force. Brisk load changes occurred at the lower extremity between the foot contact at the start of FDS and the vGRF_max_, with the latter corresponding to forces generated by knee extension during weight acceptance [22].

We observed an initial coordination of vGRF, grip force and load force, all of which decreased during the start of the first swing. In the middle of the first swing, while the body was still descending and the vGRF value was less than the weight of the subject, load force and grip force became dissociated from vGRF. The load and grip force values began to increase, probably in anticipation of the brisk changes in force that would occur during FDS. Grip and load force remained coordinated until vGRF_max_, when dissociation in the maxima was observed. Although dissociated from GF_max_, LF_max_ was coordinated with vGRF_max_. In addition, we observed an unexpected gender-dependent strategy while performing the step-down task. Compared to men, women had larger vertical displacements and higher vGRF values after the first foot contact, with lower GF/LF ratio values.

Early coupling among grip force, load force and vGRF during the start of the first swing demonstrates the predictive control of the GF, which was adapted to LF, which, in turn, was associated with vGRF. Such adaptation of grip force to load force during object transport has been demonstrated in upward and downward movements of a handheld object [12], as well as during walking while transporting an object [15–17]. This result is also in agreement with experiments where grip force adapted to load force in vertical rhythmic and discrete movements [23–25].

From the middle of the first swing, grip force and load force remained coupled, but no longer were synchronized with vGRF. While the body was still descending and the vGRF value remained below the weight of the subject, grip force and load force began to increase progressively in anticipation of the brisk changes in force that would be induced by foot contact and knee extension. Anticipatory increases in grip force and load force began approximately 100ms before foot contact and 220ms before knee extension (vGRF_max_). Similar delays have been reported in the predictive control of grip-lift synergy during brisk load increases. For example, Johansson and Westling reported a delay in the grip force increase of 150ms as a preparatory action during a task of dropping balls into a target cup attached to the bottom of a handheld object [26]. Bleyenheuft et al. observed that grip force peaked 280ms after collisions during rapid total mass increases of an object through dropping an additional mass attached to the object [3].

It has been suggested that these anticipatory actions are intended to minimize the disturbances on the rest of the body resulting from an action of one part of the body. They result from the ability of a subject to integrate, in his/her internal model, the temporal relationship between actions and their consequences [1]. Therefore, we propose that in this task, the predictive increases in grip and load force act to smooth the sharp load force increases that could be induced by brisk load changes, as observed in vGRF at the start of foot contact (FDS) and subsequent knee extension (vGRF_max_). Without anticipatory actions, a large sharp increase in load force without prior grip force increase could have led to a slip and/or loss of the object. The predictive increase in load force was induced by a relative upward movement of the hand while the body was still descending, as highlighted by the smaller displacement of the hand on the kinematic traces. This dynamic-kinematic relationship between arm movement and precision grip forces was described in the early nineties by Flanagan and colleagues whom demonstrated that the load force traces increased and decreased according to the upward and downward movement of the arm, respectively [2, 23]. Our observations on the traces of the upper extremity are similar to those observed by Flanagan. In addition, anticipatory movements leading to a load force increase before a brisk load change were previously observed in passive self-triggered collisions, particularly in paradigms where blank trials were applied [3].

Interestingly, although vGRF_max_ and LF_max_ occurred in the same time window (showing a transmission of forces generated by the lower extremity to the object held in the hand), the grip-load force coupling at this time point was dissociated. GF_max_ occurred an average of 73 ms after the load force/vGRF peak. This dissociation between LF_max_ and GF_max_ in the context of a brisk load change closely matches the desynchronization between grip force and load force peaks observed by White et al. (2011) [12] during brisk load increases induced on a handheld object during collisions in a downward direction. In this paradigm, the authors observed a progressive increase in grip force in anticipation of the impact, with GF_max_ occurring 65 ms after the collision (LF_max_). This grip force behaviour (i.e. increasing after a brisk load increase and peaking after the load force peak) was also observed in passive self-triggered collisions [3, 26] and in collisions produced when a handheld object was hit by a pendulum [9].

Grip force peaked after the collision and, thus, after the time of maximal risk of slippage. This desynchronization has been explained as a strategy to decrease the stiffness of the contact between the hand and handheld object [3, 9, 12]. The strategy provides the functional advantages of absorbing vibrations at the moment of the brisk load increase and stabilizing the hand against subsequent transients in load force by increasing grip force to its maximum [12]. This explanation is supported by the works of Lacquaniti et al. on stiffness and damping during object catching [27, 28]. Our observation of the desynchronization mechanism demonstrates that the internal model of precision grip is able to integrate a brisk load change, whatever its origin, and regulate the forces to provide an ideal grip force to dampen the load increase and secure the object.

Finally, as shown by Bleyenheuft and colleagues, if a brisk load change is expected but does not occur, the grip force increase after expected load force increase is still present, demonstrating the predictive nature of this component [3]. This adaptation of grip force to a brisk load force change, reaching a maximum after the load force change, allows ensuring damping at the moment of the brisk load change and a secure grip—to avoid slippage—afterwards [12].

Interestingly, while the time course of the predictive strategy was similar between men and women in the small sample, we observed significant differences in the forces and kinematics. The kinematic results highlighted larger vertical displacements of the body and hand after the first foot contact for women, suggesting more knee flexion. The dynamic results in women were congruent with the kinematic results, showing higher vGRF values after the first foot contact (suggesting a more important damping at landing), associated with the use of lower grip force. In contrast, men showed consistently higher values of grip/lift coupling, with less damping (i.e. higher body and hand positions after the first foot contact, with less total vertical displacement). These results suggest that in this group of 6 males and 6 females we observed the use of different strategies depending on gender, which may be explained by different hypotheses.

It may be that the grip-load force coupling relates to natural differences in locomotion between men and women. Reports have highlighted many gender differences during locomotion, including differences in gait speed [29] and running [30], with men exhibiting a higher velocity than women. Gait is more variable in men compared to women during a dual task [31], and women present a longer stride duration than men during stair descent [32]. Women, when ageing, report less self-confidence and greater cautiousness than men in their ability to manage stairs [33]. If locomotion on stairs is systematically different for men (i.e. higher speed and shorter stride) compared to women, it may be that men have adapted towards higher levels of grip force. This hypothesis is supported by the difference in the GF/LF ratio observed at the start of our traces (i.e. before starting the task).

Other potential explanations for gender differences may be related either to the dual aspect of the task (men and women are managing them differently [29, 34]) or to developmental differences learned during childhood depending on type of game mainly used [35, 36]. However, while we normalized subjects for weight, we cannot exclude non-linearities between the body-mass and the GF/LF variabilities. Therefore we cannot extrapolate these gender differences to the entire male and female population. To allow such an extrapolation future studies should be performed with a wider sample and subjects with different genders matched in body mass and other anthropomorphic parameters.

## Conclusions

Descending a single step generates brisk increases in dynamics between foot contact at the start of FDS and vGRF_max_ at knee extension. While holding a handheld object, the brisk increase at foot contact is preceded by parallel increases of load force and grip force, which probably allow smoothing of the brisk increase in dynamics. Parallelism between grip force and load force is disrupted when the forces reach their maxima; GF_max_ occurs later, probably to dampen the constraints at LF_max_ and provide a secure grip afterwards. This finding demonstrates the ability of feedforward precision grip control to include a lower extremity-induced brisk load increase. Interestingly, dynamic differences were observed between men and women, with men using systematically higher forces to hold the object. These differences were coupled to a difference in kinematics, highlighting a gender-specific strategy.
